# The role of patient age in fertility preservation among women with breast cancer: biological mechanisms and clinical implications

**DOI:** 10.1007/s10815-026-03923-0

**Published:** 2026-06-11

**Authors:** Martina Placidi, Manuela Ludovisi, Laura Pizzorno, Tina Sidoni, Giovanna Di Emidio, Carla Tatone

**Affiliations:** 1 Department of Life Science, Health, and Health Professions, Link Campus University, Rome, Italy; 2 Department of Life, Health and Environmental Sciences, University of L’Aquila, L’Aquila, Italy; 3 Breast Unit, San Salvatore Hospital, L’Aquila, Italy; 4 Medical Oncology, San Salvatore Hospital, L’Aquila, Italy

**Keywords:** AMH, Breast cancer, Chemotherapy, Endocrine therapy, Fertility preservation, Oncofertility, Ovarian reserve, Patient age

## Abstract

**Background:**

Breast cancer (BC) is the most common malignancy among women of reproductive age. Improved survival has shifted clinical focus toward survivorship and fertility preservation. While the gonadotoxic effects of anticancer therapy are well recognized, the influence of patient age on fertility outcomes remains insufficiently integrated into clinical decision-making.

**Methods:**

This review synthesizes current evidence from clinical, molecular, and translational studies addressing the relationship between age, ovarian reserve, and reproductive outcomes in women with BC undergoing gonadotoxic treatments. It also examines how age interacts with BRCA mutations, endocrine therapy, and fertility preservation strategies.

**Main findings:**

Age critically modulates the ovarian response to chemotherapy, the extent of oocyte depletion, and recovery of ovarian function. Younger patients show higher resilience and better success with fertility preservation methods such as oocyte, embryo, or ovarian tissue cryopreservation. Conversely, older women face accelerated follicular depletion, reduced AMH levels, and delayed conception due to prolonged adjuvant therapy. BRCA-related DNA repair defects further exacerbate age-related decline.

**Conclusion:**

Patient age represents the most decisive factor guiding fertility preservation in BC care. Incorporating age-specific counseling and early referral into multidisciplinary oncofertility practice is essential to optimize reproductive outcomes and align survivorship with future family planning goals.

## Introduction

Breast cancer (BC) is the most prevalent malignancy among women globally, with a rising incidence in women of reproductive age, especially those between 30 and 45 years old [1]. Approximately 4% of invasive BC cases occur in women under the age of 40 [2]. Between 2017 and 2021, the annual age-adjusted incidence rate of new BC cases among women was estimated at 129.4 per 100,000 individuals, with approximately 100 of these representing early-stage BC diagnoses [3]. Encouragingly, advances in early detection and treatment have contributed to a 1.2% annual reduction in the age-adjusted mortality rate from 2013 to 2022, resulting in a five-year relative survival rate of 91.2% as of 2020 [4]. Among premenopausal women diagnosed with BC, the five-year survival rate is 88%, compared to 91% in women aged 45–54 and 92% in those aged 65–74 [4]. With survival rates improving, there is increasing focus on post-treatment quality of life, particularly on the issue of fertility.

Chemotherapy has long been recognized for its detrimental impact on female fertility. Since the 1970 s, studies have shown that exposure to anticancer agents can lead to oocyte depletion and, in severe cases, complete ovarian failure. Due to the gonadotoxicity of individual agents or combination regimens, women treated with chemotherapy are significantly less likely to conceive compared to the general population. In this regard, an interdisciplinary area, oncofertility, that combines oncology and fertility has emerged. Fertility preservation (FP) encompasses a range of medical interventions aimed at maintaining reproductive potential before or during gonadotoxic cancer treatments. These include cryopreservation of oocytes, embryos, or ovarian tissue, and the use of gonadotropin-releasing hormone (GnRH) analogs to protect ovarian function [5]. These strategies are especially relevant for premenopausal women whose fertility may be compromised by cancer therapy, many of whom hope to conceive following recovery. Among the molecular subtypes of BC, hormone receptor-positive (HR+) disease is particularly relevant in this context, as it is more prevalent among younger women [6] and accounts for approximately 70% of cases in the premenopausal population [7].

This review addresses the impact of anticancer treatments on reproductive health and explores various FP strategies to offer personalized, optimal care for each patient considering the impact of patient age as key factor. Indeed, patient age represents one of the most critical determinants of reproductive outcomes after cancer therapy, influencing both ovarian reserve resilience and the success of fertility preservation strategies. Therefore, this review specifically focuses on age as a pivotal element guiding fertility preservation counseling and clinical decision-making.

## Impact of age on female fertility and ovarian reserve

The female reproductive system ages more rapidly than any other system in the human body. Research and population studies have shown that a woman’s biological ability to conceive declines with age, similar to patterns observed in other species [8]. Female fertility peaks in the 20s, declines significantly in the late 30s, even when menstrual cycles remain regular, and ends with menopause around the average age of 50–51 years [8]. In recent years, the age at which women have their first child has risen markedly. Several studies suggest that this trend may be attributed to the increasing participation of women in higher education and the workforce, as well as to their pursuit of financial stability [9]. These factors often lead women to postpone motherhood until later in life. According to Eurostat, the average age of first-time mothers in Europe reached 29.8 years in 2023, representing a substantial increase compared to previous decades [10]. This rise in maternal age is closely linked to issues of infertility, as a woman’s natural ability to conceive declines with advancing age [9]. Evidence indicates that the optimal age range for achieving conception and ensuring a healthy pregnancy is between 20 and 34 years. After the age of 35, fertility rates drop sharply, and achieving pregnancy becomes increasingly challenging [9]. Data from over 120,000 assisted reproduction technology (ART) procedures in the USA show that the success rate per embryo transfer drops significantly with age: from 43.2% in women under 35 to just 5.9% in those over 42 [11]. By the fifth month of fetal development, the ovarian follicle pool contains around 7 million oogonia, but most undergo atresia. The surviving oogonia enter meiotic prophase I, becoming primary oocytes surrounded by epithelial cells that form primary follicles [12]. These oocytes remain arrested in the diplotene stage until puberty. At birth, about 1 million primordial follicles are present; this number drops to around 300,000 by menarche [12]. The existence of postnatal oogenesis in humans remains unproven; however, the scientific community supports the possibility of limited oocyte renewal occurring during a woman’s lifetime [13]. As the number of oocytes decreases, their quality also tends to deteriorate. Oocyte quality, often referred to as developmental competence, reflects the ability of an oocyte to complete meiosis, undergo successful fertilization, and support early embryonic development. Although much of the complex mechanism underlying the decline in oocyte quality remains to be fully elucidated, several key factors appear to play crucial roles in this process. These include mitochondrial dysfunction, spindle assembly defects, loss of cohesion, telomere shortening, and macromolecular damage, all of which contribute to an increased risk of chromosomal nondisjunction and, consequently, oocyte aneuploidy with advancing maternal age [14]. Given these patterns, several key considerations must be made when studying the cellular and molecular biology of oocytes and follicles. First, the oocytes of primordial follicles remain quiescent for up to 40 years, yet oocytes are vulnerable to damage. Second, the development of a mature oocyte relies on intricate signaling between the different ovarian compartments [12]. Since infertility with age is multifactorial and the biology of folliculogenesis is complex, understanding ovarian aging remains particularly challenging. Nevertheless, some insights can be drawn from general aging theories. The most prominent theory of ovarian aging involves the diminished ability of oocytes and surrounding cells to neutralize reactive oxygen species (ROS), a primary contributor to age-related cellular damage [14]. Loss of DNA repair capability, mitochondrial dysfunction, and reduced bioenergetics are also believed to underlie oocyte aging [14]. Research shows that during reproductive aging serum levels of follicle-stimulating hormone (FSH) rise particularly during the early follicular phase contributing to the accelerated depletion of the follicular reserve. However, studies using transgenic mouse models with elevated FSH levels demonstrate that while the proportion of fertilizable oocytes decreases, the overall size of the follicular pool remains relatively unchanged, suggesting a complex and multifaceted role of FSH in ovarian aging [15].

Anti-Müllerian hormone (AMH), a 140 kDa glycoprotein secreted by granulosa cells of developing, non-dominant follicles, operates independently of the pituitary–gonadal axis. AMH serum levels closely correlate with the number of antral follicles and provide a reliable biomarker for assessing ovarian reserve and the quantity of remaining primordial follicles. Currently, AMH is considered the most effective clinical marker for evaluating ovarian reserve and is also useful for predicting reproductive lifespan, diagnosing ovarian dysfunction, and assessing the effects of gonadotoxic treatments or surgical interventions [16]. Traditionally, it has been believed that the irreversible decline in ovarian reserve in mammals is due to the absence of proliferative germ cells in adult ovarian tissue. However, emerging research in both rodents and humans suggests the existence of germline stem cells, capable of generating new oocytes and forming follicles in adult ovaries a process termed neo-oogenesis [17]. The progressive depletion of the primordial follicle pool during physiological ovarian aging has important implications in the oncologic setting. Age-related declines in AMH levels and reduced inhibition of follicular activation create a biologically vulnerable environment in which chemotherapy-induced accelerated follicle recruitment (“burn-out” effect) may further exacerbate follicular depletion, as discussed in subsequent sections. Overall, these considerations highlight that age is not only a marker of baseline reproductive capacity but also a key modifier of treatment-related ovarian damage, directly influencing fertility preservation strategies and long-term reproductive outcomes in breast cancer patients. These biological mechanisms provide the basis for understanding the differential impact of systemic cancer treatments on ovarian function across age groups.

## Effects of BRCA mutations on ovarian reserve

Genetic or hereditary factors, including BRCA1/2 pathogenic variants, account for approximately 5–10% of BC cases overall. BRCA genes play a key role in the ATM-mediated DNA double-strand break repair pathway. Loss of BRCA function leads to impaired DNA repair, causing genomic instability that promotes cancer development, particularly early-onset breast and ovarian cancers [18]. In oocytes, accumulated DNA damage over time contributes to age-related decline in oocyte quality, reduced fertility, and the natural onset of infertility approximately a decade before menopause [19]. From a biological perspective, defective DNA repair in BRCA pathogenic variant carriers may accelerate follicular loss, potentially leading to diminished ovarian reserve and premature ovarian insufficiency (POI). High BRCA gene expression in germ cells and embryos further supports a role in gametogenesis and embryogenesis, suggesting possible implications for reproductive potential [19]. However, clinical evidence on the impact of BRCA pathogenic variants on ovarian reserve and fertility remains inconsistent. Some studies report lower AMH levels or reduced ovarian response, particularly in BRCA1 carriers, whereas others show no significant differences. Findings regarding age at menopause are also heterogeneous. Importantly, recent large-scale analyses have demonstrated a strong association between younger age at BC diagnosis and the presence of BRCA1/2 pathogenic variants, particularly for BRCA1, while this association is less pronounced for other susceptibility genes [18]. These findings support the concept that BRCA-related BCs are more likely to occur at younger ages, indirectly reinforcing the clinical relevance of age in this subgroup. BRCA1 pathogenic variant carriers have been reported to exhibit significantly lower AMH levels and reduced ovarian reserve compared to age-matched non-carriers [20], likely due to impaired DNA repair mechanisms accelerating follicular depletion. While BRCA2 carriers may experience less pronounced effects, some studies have also reported altered ovarian function in this group [21]. Furthermore, BRCA pathogenic variant carriers are frequently diagnosed with breast cancer at a younger age and may undergo risk-reducing bilateral salpingo-oophorectomy, further narrowing their reproductive window. These factors highlight the importance of timely fertility counseling and intervention. Despite these considerations, fertility preservation strategies such as oocyte cryopreservation and ovarian tissue cryopreservation remain feasible and effective in BRCA pathogenic variant carriers, although oocyte yield may be lower. Ovarian tissue transplantation should be approached cautiously due to the theoretical risk of reintroducing malignant cells, particularly in BRCA-associated cancers [22]. Overall, healthcare providers must carefully balance oncologic safety with reproductive planning and ensure that BRCA carriers receive individualized, early oncofertility counseling and access to appropriate fertility preservation strategies.

## Mechanisms of chemotherapy-induced ovarian damage: cellular and molecular insights

The treatment strategy for BC is influenced by tumor subtype, stage, germline BRCA pathogenic variant status, patient age, and menopausal state. Systemic treatments include chemotherapy, endocrine therapy, targeted agents, and immunotherapy [19], all contributing to the risk of treatment-related amenorrhea (TRA) and POI [20]. Chemotherapy-induced gonadotoxicity has been recognized for decades [21], with treated women showing a 13–38% reduction in post-treatment fertility compared to the general population [22]. The extent of ovarian damage depends on treatment type and dose, as well as baseline ovarian reserve and patient age [22, 23].

**Fig. 1:**
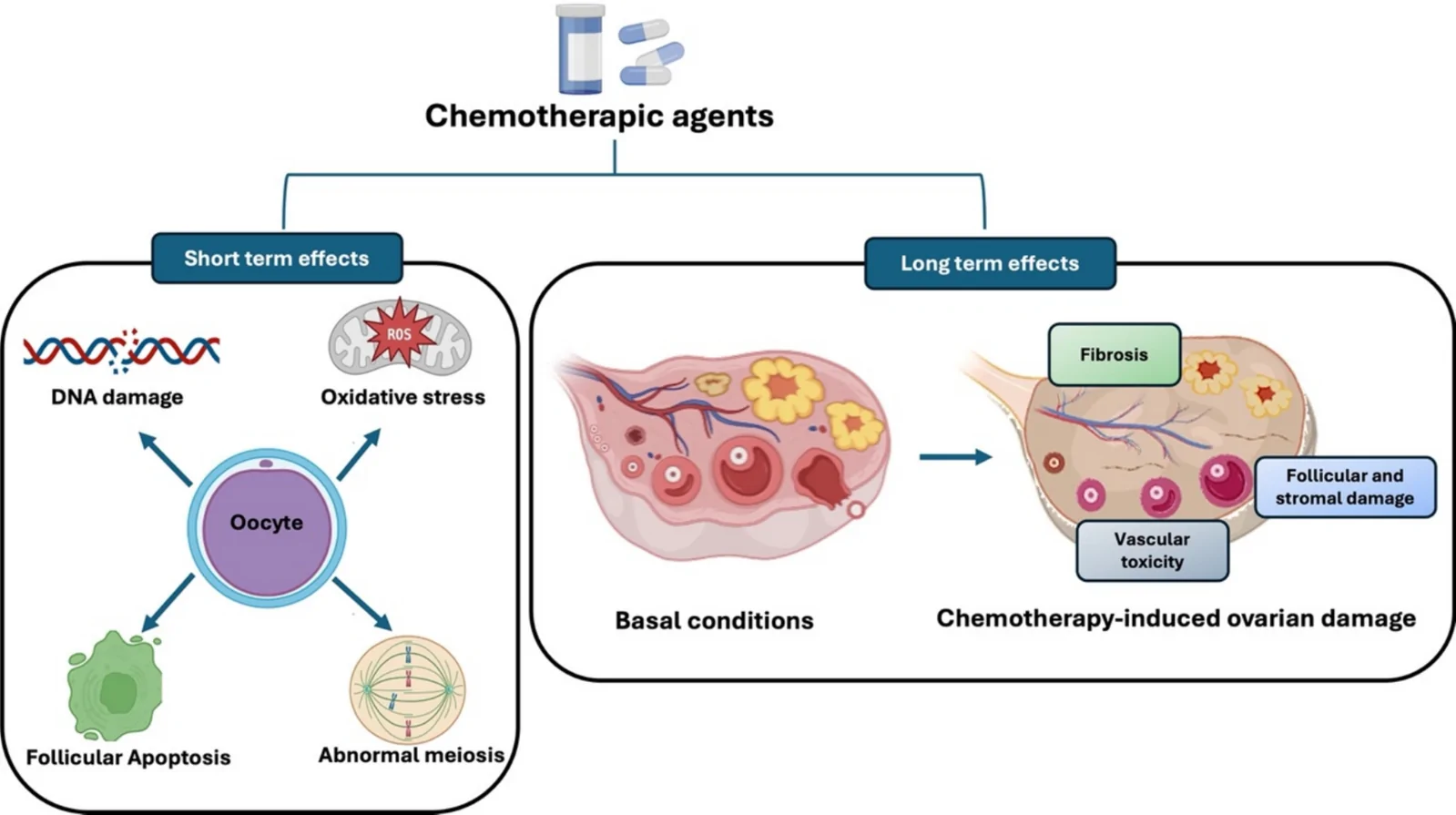
Schematic representation of the short and long-term effects of gonadotoxic drugs on the ovary

Chemotherapy affects the ovary through both cell cycle–dependent (e.g., antimetabolites) and independent mechanisms (e.g., alkylating agents, anthracyclines, and platinum compounds), targeting oocytes, granulosa cells, and stromal tissue [24]. The effects on ovarian function can be categorized into short-term and long-term consequences (Fig. 1). Short-term effects primarily involve depletion of growing follicles, leading to acute amenorrhea [25], and are mainly driven by DNA damage, including double-strand breaks induced by alkylating agents and anthracyclines, and DNA crosslinking caused by platinum compounds, resulting in follicular apoptosis [26]. Additional mechanisms include oxidative stress and mitochondrial dysfunction (anthracyclines), microtubule disruption (taxanes), and inhibition of nucleic acid synthesis (antimetabolites).

Damage to proliferating granulosa cells further impairs follicular maturation, while stromal injury contributes to reduced estradiol production. Similar molecular signatures of ovarian injury, including activation of apoptotic and inflammatory pathways, have also been described in non-oncologic conditions [27, 28].

Long-term effects are mainly driven by depletion of the primordial follicle pool, leading to reduced ovarian reserve, POI, and infertility [29]. This damage is mediated by two main mechanisms: direct DNA damage to dormant follicles and accelerated follicle recruitment (“burn-out” effect) [22]. In the latter, loss of growing follicles triggers activation of primordial follicles via the PI3K signaling pathway, potentially mediated by reduced AMH levels, leading to loss of follicular quiescence and increased vulnerability to subsequent chemotherapy cycles [22].

In addition, chemotherapy induces vascular and stromal injury, including ischemia, fibrosis, and inflammatory damage, further impairing ovarian endocrine function. Agents such as vinca alkaloids and alkylating drugs may disrupt ovarian vasculature, exacerbating follicular loss [23]. Importantly, the clinical impact of these mechanisms is strongly age-dependent, with older women showing increased susceptibility to chemotherapy-induced POI [22].

## Amenorrhea in breast cancer chemotherapy: risk factors and recovery

TRA occurs in up to 83.6% of BC patients receiving chemotherapy, though recovery rates vary 43.6% of patients had TRA at one year, and 18.9% at 2 years post-treatment initiation [20]. Notably, about 70% of women with TRA resumed menstruation within one year, and 90% within 2 years. However, even after menstrual recovery, ovarian reserve and fertility potential may remain significantly reduced. Importantly, resumption of menses should not be interpreted as full restoration of fertility. Menstrual recovery primarily reflects hypothalamic–pituitary–ovarian axis reactivation and does not necessarily indicate adequate ovarian reserve or preserved oocyte competence. Particularly in women over 35–40 years of age, ovarian function recovery may occur despite a critically diminished primordial follicle pool and persistently low AMH levels. Consequently, the probability of spontaneous conception may remain substantially reduced, and many patients require assisted reproductive technologies to achieve pregnancy. These observations underscore the need to clearly distinguish endocrine recovery from true reproductive potential when counseling BC survivors [20].

Risk and recovery from TRA are influenced by both patient-specific and treatment-related factors. Key patient factors include age, baseline ovarian reserve, and presence of germline BRCA pathogenic variant. Treatment factors encompass chemotherapy type, dose, and duration [30]. Age is the most critical determinant of gonadotoxicity. Women over 35 have a threefold higher risk of TRA compared to those under 30 [31]. The same chemotherapy regimen poses variable TRA risk across age groups: high risk in women ≥ 40, intermediate risk in those aged 30–39, and low risk in those < 30 [20].

Women with BRCA1/2 pathogenic variant represent a unique subgroup. These mutations impair homologous recombination DNA repair, increasing susceptibility to DNA damage [34]. From a biological and mechanistic perspective, BRCA pathogenic variants may contribute to accelerated follicular depletion due to impaired DNA repair capacity in oocytes, potentially leading to reduced ovarian reserve and earlier menopause. Consistently, BRCA carriers have been reported to experience menopause 1–3 years earlier and may exhibit lower oocyte yield during fertility preservation procedures. However, clinical evidence remains heterogeneous and inconclusive. While some studies suggest lower AMH levels in BRCA carriers, others report no significant differences in ovarian reserve markers or response to ovarian stimulation. Importantly, available clinical data indicate that BRCA mutation status does not appear to significantly modify the risk of TRA or AMH decline following chemotherapy compared to non-carriers [35].

Furthermore, as highlighted in recent systematic reviews on fertility and systemic therapies in early BC, robust prospective data on ovarian function and fertility outcomes in BRCA carriers are limited, and the independent contribution of genetic background versus treatment exposure remains difficult to disentangle [32].

TRA in BC patients has been extensively studied, with key agents such as cyclophosphamide, doxorubicin, and taxanes being most strongly associated with fertility impairment. The gonadotoxic impact of the main systemic BC treatments across different age groups is summarized in Table 1.

**Table 1:** Gonadotoxic effects of breast cancer systemic treatments across different female age groups

Treatment agents	Mechanism of action	Effects in prepubertal girls	Effects in women ≤ 40 years of age	Effects in women > 40 years of age
Alkylating agents (e.g., cyclophosphamide)	DNA crosslinking; double-stranded DNA breaks; primordial follicles damage; accelerated follicle recruitment (“burn-out”)	High	Moderate to high	Very high
Anthracyclines (e.g., doxorubicin)	DNA intercalation; topoisomerase II inhibition; oxidative stress → oocyte mitochondrial damage	Low-moderate	Moderate	High
Taxanes (paclitaxel, docetaxel)	Microtubule stabilization → spindle dysfunction in mature oocytes; affects growing follicles	Low-moderate	Moderate	High
Platinum compounds	DNA crosslinking; induces apoptosis in growing follicles; accelerate primordial follicle recruitment	Low-moderate	Moderate	High
Antimetabolites	Inhibition of DNA/RNA synthesis; damage to proliferating granulosa cells and growing follicles	Low	Low-moderate	Moderate
Tamoxifen	SERM (Selective Estrogen Receptor Modulator); estrogen receptor blockade in breast; non-cytotoxic to follicles	Not used	Very low	Low-moderate
Aromatase inhibitors (with ovarian function suppression)	Suppression of systemic estrogen production	Not used	Very low	Low-moderate
GnRH agonists	Pituitary GnRH receptor downregulation → LH/FSH suppression → transient ovarian quiescence; mitigates chemotherapy-induced gonadotoxicity	Not used	Very low/potential protective	Low
Anti-HER2 Therapy (Trastuzumab, pertuzumab, T-DM1, T-DXd)	HER2 inhibition; no direct ovarian cytotoxicity; possible protection of ovarian vasculature (preclinical)	Not used	Very low	Low-moderate
PARP inhibitors	Inhibition of DNA repair; reduction of primordial follicles and impairment of granulosa cell function (preclinical models)	No data	Uncertain/potentially moderate	Moderate-high
Immune checkpoint inhibitors (pembrolizumab, ipilimumab)	Immune activation; immune-mediated endocrinopathies (hypophysitis, hypothyroidism) → secondary hypogonadism; possible ovarian dysfunction	No data	Moderate	Moderate-high

### Alkylating agents

Among chemotherapeutic agents, cyclophosphamide, an alkylating agent, has the highest gonadotoxic potential, doubling the risk of TRA. Its mechanism likely involves direct damage to the non-growing primordial follicle pool, the basis of ovarian reserve [30]. Consistently, AMH levels remain lower post-treatment in patients exposed to alkylating agents [33]. Doxorubicin (an anthracycline) and taxanes (e.g., docetaxel and paclitaxel) also impair ovarian function but with lower risk than cyclophosphamide. Overall, chemotherapy protocols that include alkylating agents such as cyclophosphamide are associated with greater reductions in AMH levels and poorer post-treatment recovery compared to regimens without alkylating agents [33].

### Hormone therapy

Tamoxifen (TAM) is the most established adjuvant endocrine therapy for both premenopausal and postmenopausal patients [34]. TAM is associated with an increased risk of TRA, although its effects are largely reversible.

Recent studies indicate that temporarily interrupting TAM therapy may be a viable option for carefully selected patients wishing to conceive, showing high pregnancy success rates and comparable short-term recurrence outcomes. Additionally, emerging evidence of TAM’s potential ovarian-protective properties provides a more nuanced understanding of its role in fertility preservation [35]. Nevertheless, important challenges persist particularly concerning teratogenic risks, optimal timing for therapy interruption, and global accessibility of fertility preservation services. In the ASTRRA study, which followed patients who recovered from chemotherapy-induced TRA and subsequently received TAM (with or without goserelin), 69% resumed menstruation, including 91% of women under 35 [36]. Most also recovered normal levels of FSH (98%) and estradiol (74%) [36]. Over a 5-year period of TAM therapy following chemotherapy, menstruation resumed in approximately two-thirds of patients, particularly among those under 35 years of age. Younger age, treatment with adriamycin without taxanes, and shorter chemotherapy duration were associated with better long-term preservation of ovarian reserve [36]. Long-term follow-up and large-scale studies are needed to confirm the long-term safety and effectiveness of this approach.

### Anti-HER2 therapy

In HER2-positive BC, treatment involves anti-HER2 agents such as trastuzumab, pertuzumab, lapatinib, ado-trastuzumab emtansine (T-DM1) and trastuzumab deruxtecan (T-DXd) [34]. Data from the ATTEMPT trial showed significantly lower TRA rates at 18 months in patients treated with T-DM1 compared to those who received trastuzumab plus paclitaxel suggesting a more favorable gonadal toxicity profile with anti-HER2 monotherapy [37]. The ALTTO trial actually showed that dual anti-HER2 therapy did not significantly increase the risk of TRA compared to monotherapy [38]. Other studies have found that trastuzumab combined with chemotherapy may help protect ovarian function, leading to higher AMH recovery rates compared to chemotherapy alone [39]. Preclinical findings suggest that HER2 inhibition may protect against chemotherapy-induced gonadotoxicity, possibly due to HER2 expression in ovarian vasculature [20].

### Targeted therapies

Targeted cancer therapy is collectively made up of two classes: immunotherapy and small molecule inhibitors [40]. Immunotherapy has revolutionized cancer treatment, with checkpoint inhibitors (CPIs) such as pembrolizumab now approved for early-stage triple-negative breast cancer, as demonstrated in the KEYNOTE-522 trial. This is particularly relevant for younger women, who have a higher incidence of triple-negative disease [41]. However, the effects of CPIs on fertility and pregnancy remain insufficiently characterized. Current evidence is limited to preclinical data, case reports, and adverse event analyses from clinical trials. Fetal risks are thought to result primarily from maternal immune activation rather than direct cytotoxicity; therefore, the National Comprehensive Cancer Network recommends effective contraception during CPI therapy and for at least 5 months after the final dose [42]. Immune-related endocrinopathies represent a key concern. Although primary hypogonadism induced by CPIs is rare, secondary hypogonadism due to hypophysitis and hypothyroidism is more frequent. Pituitary dysfunction leads to reduced secretion of FSH and LH, potentially resulting in anovulation or premature menopause [42].

CDK4/6 inhibitors, such as palbociclib, ribociclib, and abemaciclib, act by blocking cell-cycle progression from the G1 to S phase through inhibition of retinoblastoma protein phosphorylation. These agents are approved for advanced hormone receptor–positive, HER2-negative BC. Preclinical studies in rats exposed to high doses of palbociclib have shown no adverse effects on fertility. However, clinical data remain limited. Given the 2–3-year duration of adjuvant therapy, during which conception must be avoided, CDK4/6 inhibitors may indirectly reduce fertility potential by delaying pregnancy attempts [43].

PARP inhibitors, such as olaparib, may exert direct gonadotoxic effects. Preclinical studies have demonstrated a reduction of approximately 36% in primordial follicle count in mice exposed to olaparib, without affecting growing follicles, suggesting a selective depletion of dormant oocytes. Additionally, olaparib has been shown to impair granulosa cell function, leading to reduced estradiol production and compromised follicular support. Partial recovery of ovarian activity following short-term discontinuation has also been observed in animal models; however, the long-term reproductive impact of prolonged PARP inhibitor exposure remains uncertain [43]. In contrast, clinical evidence in humans remains limited. To date, no robust prospective data have conclusively demonstrated a direct detrimental effect of PARP inhibitors on ovarian reserve or fertility outcomes, and available evidence is often confounded by concomitant chemotherapy exposure and patient age. Recent clinical studies evaluating ovarian reserve in young BC patients receiving systemic therapies further highlight the difficulty in isolating drug-specific effects, as substantial declines in AMH are primarily driven by chemotherapy rather than adjunctive treatments [44]. Therefore, the current evidence on PARP inhibitor–related gonadotoxicity should be interpreted with caution, and dedicated prospective studies are needed to clarify their long-term reproductive safety.

Immune checkpoint inhibitors (ICIs), particularly pembrolizumab, are increasingly incorporated into early-stage triple-negative breast cancer regimens. Their reproductive effects remain poorly characterized, complicating fertility counseling. Clinical evidence in breast cancer patients is currently limited, and most concerns arise from immune-related endocrinopathies, which may lead to persistent hormonal dysfunction, particularly in younger women.

Available human data are scarce and largely derived from small observational studies. In non-BC populations, including melanoma patients, limited evidence suggests post-treatment reductions in estradiol, luteinizing hormone, and AMH following ipilimumab therapy, raising the possibility of impaired ovarian reserve [46]. However, these findings are based on small cohorts and may be confounded by prior or concomitant treatments. Mechanistic and indirect evidence further supports a potential impact on reproductive function, primarily mediated through immune-related effects on the hypothalamic–pituitary–gonadal axis rather than direct ovarian toxicity. Importantly, as highlighted in recent reviews of targeted therapies in BC, including PARP inhibitors and immunotherapy, robust clinical data on ovarian function and fertility outcomes are lacking, as these endpoints are rarely included in clinical trials [45]. Overall, the current evidence remains insufficient to draw definitive conclusions, and prospective studies are needed to better define the long-term reproductive safety of ICIs.

## Methods for fertility preservation

FP is increasingly recognized as an essential component of cancer treatment rather than an optional consideration. The American Society of Clinical Oncology (ASCO) recommends that patients be promptly referred to a fertility specialist before starting cancer therapy [5].

**Fig. 2:**
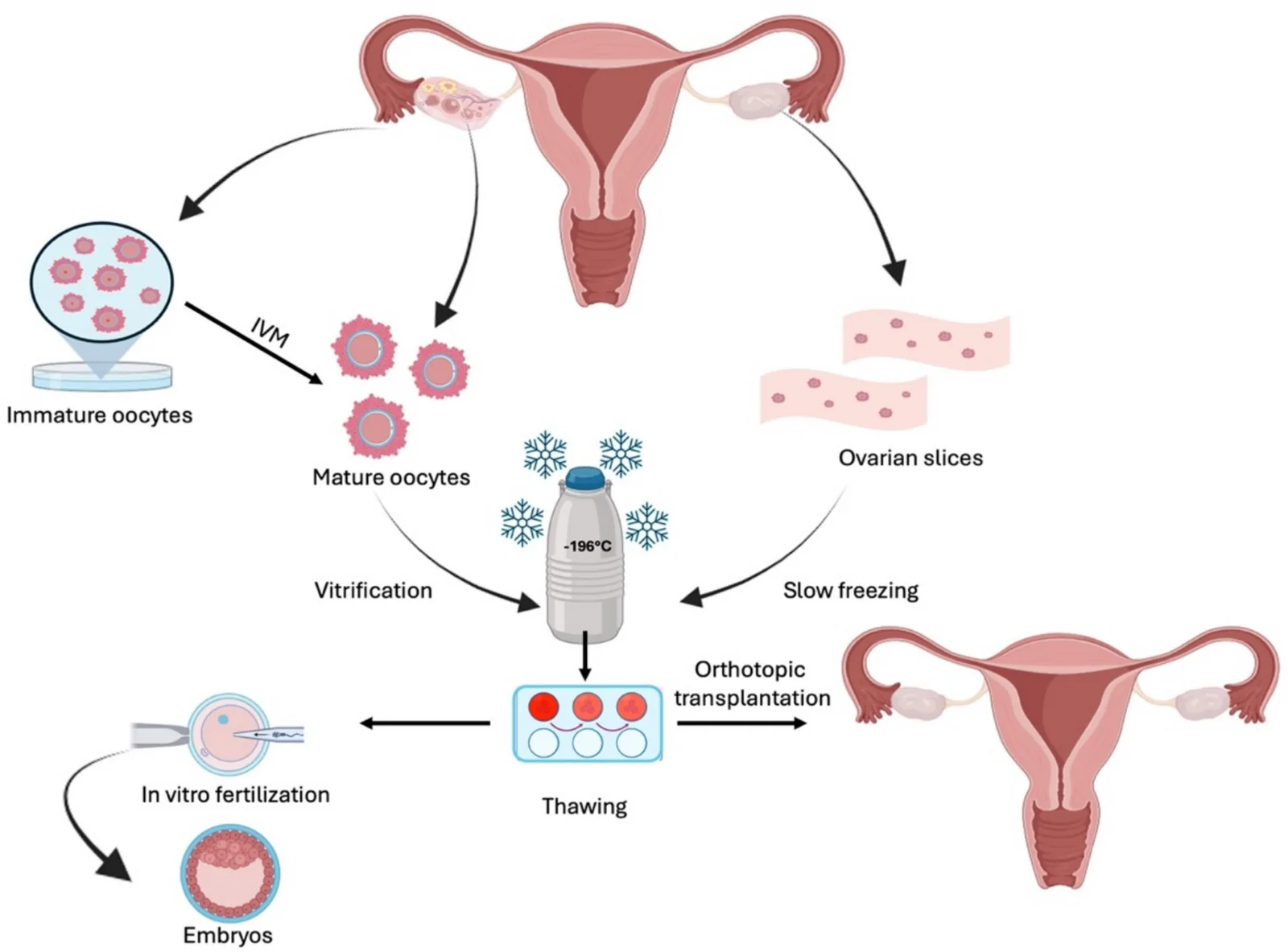
Options for female fertility preservation

Choosing the best FP approach requires considering multiple factors such as the patient’s age, cancer stage and severity, urgency of treatment, desire for future pregnancy, and the available time between controlled ovarian stimulation (COS) and cancer therapy initiation. Several FP options exist, and decisions, whether to cryopreserve oocytes, embryos, or ovarian tissue, or to administer GnRH agonist (GnRHa) alongside systemic therapy, should be individualized based on the patient’s medical condition, needs, and goals [20] (Fig. 2).

Embryo cryopreservation is generally preferred for patients with a male partner, as it is a well-established technique offering a high chance of FP, whereas oocyte cryopreservation is typically chosen by patients without a male partner. Both methods are clinically validated and widely practiced. Female age plays a decisive role in the success of oocyte cryopreservation. Younger women tend to have a higher ovarian reserve and produce more mature oocytes per stimulation cycle, leading to increased chances of eventual pregnancy. Numerous studies have demonstrated high oocyte survival, fertilization, pregnancy rates, and live birth rates decline progressively with increasing maternal age at the time of oocyte cryopreservation, while the number of thawed oocytes required to achieve a single live birth rises significantly with age. As live birth rate is strongly associated with the age of the oocyte, current recommendations advise performing oocyte cryopreservation before 35 years of age. In contrast, outcomes are generally poor when cryopreservation is undertaken after the age of 40 [46]. In BC populations, outcomes have been comparable to non-cancer controls when stimulation is performed with protocols that incorporate TAM or letrozole to minimize estrogen exposure [47]. However, success is closely tied to age and ovarian reserve at the time of preservation, and delays in referral may result in fewer retrievable oocytes and diminished outcomes. Moreover, some patients may require more than one cycle to achieve an optimal yield, which necessitates coordination with oncology teams to avoid delaying systemic therapy [47]. These age-related differences underscore the importance of early FP planning in young BC patients to maximize the likelihood of future reproductive success. COS is performed to maximize the number of oocytes or embryos for freezing, thereby improving chances of conception. COS and oocyte retrieval usually require 10 to 12 days and must be completed prior to starting systemic anticancer therapy. Early referral to a FP center is crucial to ensure patients are well-informed about their options and to minimize delays in cancer treatment [20]. The ovarian stimulation protocol is aimed at obtaining the highest number of oocytes within the limited time available. Initial assessment of ovarian function includes AMH, FSH, and antral follicle count, which guide the starting gonadotropin dose. While ovarian response varies with age and ovarian reserve, the target is generally 10 to 15 oocytes. Since most patients can only undergo one or two stimulation cycles before cancer treatment, choosing an effective protocol is essential. Several protocols are available as described below. Traditionally, triggering of final oocyte maturation occurs when two to three follicles reach 17 to 18 mm in diameter using hCG and/or GnRHa; however, for BC patients, triggering is often delayed until the largest follicle reaches 20 mm. Oocyte retrieval via transvaginal ultrasound guidance is performed 34 to 36 h post-triggering [48].

COS typically begins in the early follicular phase; however, cancer patients often require urgent treatment and cannot wait for menstruation. Based on the multiple follicular wave theory, COS can be initiated at any point during the menstrual cycle, known as random-start or luteal-phase stimulation, enabling timely FP without delaying therapy. Although this approach may require slightly more gonadotropins and a longer stimulation period, the number of mature oocytes retrieved is comparable to conventional protocols [48].

In BC patients, co-administration of letrozole (5 mg daily) with gonadotropins is commonly used during COS to suppress estradiol (E2) levels, minimizing hormonal stimulation of hormone-sensitive tumors. Letrozole is continued until triggering and for approximately 1-week post-retrieval, until E2 drops below 50 pg/mL. This strategy effectively reduces E2 exposure without compromising oocyte yield and has not been associated with increased cancer recurrence, including in BRCA mutation carriers [20]. Final oocyte maturation is triggered using hCG, a GnRHa, or both in combination (dual trigger). GnRHa-based triggers are associated with higher oocyte maturation and fertilization rates, as well as reduced incidence of ovarian hyperstimulation syndrome (OHSS), compared to hCG alone. However, approximately 5% of patients show suboptimal response to GnRHa, particularly those with low BMI or serum LH levels. In BC patients undergoing COS with letrozole, delaying the trigger until leading follicles reach 20 mm and using a dual trigger protocol may enhance oocyte maturation [20]. In selected cases, double stimulation, performing two COS cycles within a single menstrual cycle (one in the follicular and one in the luteal phase), can significantly increase total oocyte or embryo yield. This approach is particularly useful when time before chemotherapy is limited or when the first cycle yields an insufficient number of oocytes [49].

The retrieval procedure is identical for both oocyte and embryo cryopreservation, with in vitro fertilization (IVF) occurring in the latter. COS and oocyte retrieval are relatively safe, outpatient procedures with minimal risk. Possible complications include intraperitoneal hemorrhage from needle puncture and OHSS-related ascites. In high-risk patients, cabergoline or GnRH antagonists may be used prophylactically. Since embryo transfer is not immediate, OHSS symptoms typically resolve quickly without further risk in cancer patients [50].

Efforts to optimize oocyte yield in BC patients incorporate clinical markers such as BRCA1/2 pathogenic variant status, hormone receptor status, and blood markers. Because BRCA1/2 pathogenic variant carriers have a 10–50% lifetime risk of ovarian cancer, cryopreservation of oocytes or embryos is preferred over ovarian tissue freezing in these cases. Preimplantation genetic testing can be considered to detect BRCA1/2 pathogenic variants in embryos [20].

Although concerns exist regarding potential chemotherapy delays due to FP procedures, evidence indicates no significant delay between surgery and chemotherapy [20].

Pregnancy outcomes post-cancer treatment are limited but encouraging. Frozen embryo transfers in cancer survivors show similar cumulative pregnancy and live birth rates compared to patients with tubal infertility. Cobo et al. reported clinical pregnancy and live birth rates of 41.4% and 31%, respectively, following oocyte cryopreservation in cancer patients, with a cumulative live birth rate of 35.2% when including subsequent frozen embryo transfers [51]. Outcomes for cancer survivors appear comparable to elective FP cases, though further long-term follow-up is needed.

### Ovarian tissue cryopreservation

Ovarian tissue cryopreservation (OTC) is an established FP technique that can be offered to patients with cancer for future autologous transplantation. Because it does not require prior COS, OTC can be performed immediately, making it suitable for patients who cannot postpone chemotherapy. Moreover, as the procedure does not depend on sexual maturity, it represents the only viable FP option for prepubertal girls. In addition to fertility restoration, OTC is being explored as a means to reestablish global ovarian endocrine function. This technique may be offered either as an alternative or as an adjunct to oocyte or embryo cryopreservation. The decision to proceed with OTC should be individualized, taking into account patient preferences, clinical context, future reproductive goals, anticipated success rates, and legal or ethical considerations [5]. OTC maintains both fertility and hormonal function and does not delay chemotherapy. However, it requires laparoscopic surgery for tissue harvesting and transplantation. After cryoprotectant treatment, tissue is frozen by slow-freezing or vitrification and stored in liquid nitrogen. Transplantation may be orthotopic (OT) or heterotopic (HE). OT recreates a physiologic environment that supports follicular development by restoring paracrine signaling, vascularization, and optimal temperature conditions. This procedure enables recovery of endocrine function, allowing women either to conceive spontaneously or to undergo IVF if needed. The first live birth resulting from transplantation of cryopreserved ovarian tissue was reported in 2004 [52]. HT involves grafting ovarian tissue outside the pelvic cavity, typically into the subcutaneous tissue or along the peritoneal lining. Although follicular activity has been observed in various heterotopic sites, dominant follicle development is rare in subcutaneous grafts, as follicles often undergo atresia before reaching ovulatory size, making the retrieval of mature oocytes challenging. In contrast, the peritoneal lining represents the most favorable heterotopic site, capable of supporting follicular growth to the size required for ovulation [53]. Endocrine function restored in more than 95% of cases, with ovarian tissue maintaining hormonal activity for several years despite a limited follicular pool [54]. OTC is generally safe for BC patients except those with metastatic disease or high relapse risk. Although no consensus guidelines exist, OTC is discouraged in patients over 45 due to diminished ovarian reserve and graft quality. An ideal age range is suggested between 30 and 40 years, but decisions should be personalized [20]. Fleury et al. reviewed 16 BC patients undergoing OTC and ovarian tissue transplantation (OTT), showing graft function resuming within 1–5 months and lasting up to 15 months. No tumor cells were detected in ovarian tissue from 272 patients in seven studies, although negative tests on tissue fragments cannot completely exclude metastasis. Two BC recurrences after OTT were reported, but causality is unclear. The risk of cancer cell transmission through grafts is considered very low [55]. Clinical evidence confirms the effectiveness of OTC, particularly in restoring ovarian endocrine function and fertility, while only a limited number of pregnancies and live births have been achieved following HT [53].

Ongoing advancements in cryopreservation methods, transplantation techniques, and approaches to minimize ischemic and cryogenic injury are expected to further enhance OTC success rates. The integration of OTC into standard reproductive practice represents a promising step forward in FP. Future research should aim to refine OTC protocols, reduce tissue damage during processing and grafting, and address ethical considerations, particularly regarding its application in prepubertal girls, to ensure safe and equitable clinical implementation.

### In vitro maturation (IVM)

The term in vitro maturation (IVM) traditionally describes the process by which immature cumulus-oocyte complexes (COCs) are collected from small antral follicles and cultured from the germinal vesicle stage at prophase I through meiosis I to achieve the metaphase II stage. These COCs are typically retrieved from follicles that have not been exposed to gonadotropins or preovulatory triggers or following only minimal FSH stimulation [56].

Originally developed as a FP method for cancer patients, IVM is now being explored for a broader range of indications. Among oncology patients, BC represents the most common context for IVM. Most available data derive from young women with BC undergoing oocyte pick-up IVM or ovarian tissue oocyte IVM. These patients face a high risk of treatment-induced ovarian insufficiency, compounded by delayed or discouraged pregnancy after therapy, prolonged endocrine treatment in hormone receptor-positive cases, and hereditary cancer predisposition. As ovarian stimulation is feasible before adjuvant but less so before neoadjuvant chemotherapy, IVM has emerged as an essential emergency FP strategy for women requiring urgent treatment [56]. IVM may also benefit patients with hereditary BC syndromes requiring preimplantation genetic testing, where limited embryo availability could affect reproductive outcomes.

The combination of IVM with OTC has expanded its clinical potential. In prepubertal girls, IVM of ex vivo retrieved COCs from ovarian tissue offers a new, though still experimental, FP option, as data on oocyte developmental competence remain limited. However, only four live births after IVM for FP have been reported, and the pregnancy rates remain lower than those for oocyte and embryo cryopreservation; thus, IVM is still considered experimental [57]. Further studies with larger cohorts and long-term follow-up are necessary.

### Ovarian suppression with GnRH analogues

Endocrine therapy has markedly improved outcomes in premenopausal women with hormone receptor-positive, HER2-negative (HER2-) BC. Within this therapeutic context, ovarian function suppression is a key component of neoadjuvant and adjuvant treatment strategies. GnRHa offer a reversible, non-invasive method of ovarian suppression, with the additional potential to mitigate chemotherapy-induced gonadotoxicity. GnRHa act by binding to pituitary GnRH receptors, leading to receptor desensitization and downregulation in gonadotropic cells [58]. This process suppresses LH and FSH secretion, thereby reducing ovarian estradiol synthesis and circulating estrogen levels, ultimately inhibiting the growth of HR+ tumors. Current clinical guidelines endorse the use of GnRHa across various treatment settings for premenopausal patients with HR+/HER2- disease. However, their use in practice remains inconsistent, limited by side effects, uncertainties regarding optimal treatment duration, and lack of consensus on patient selection by recurrence risk. The use of GnRHa as part of ovarian function suppression in premenopausal women with HR+ BC continues to demonstrate significant reductions in disease recurrence and improvements in overall survival [58]. Long-term follow-up data consistently confirm these durable benefits. The addition of GnRHa to endocrine therapy is particularly beneficial for premenopausal patients at intermediate or high risk of recurrence, especially younger women, those with lymph node–positive disease, or with adverse prognostic features such as high tumor grade or large tumor size. Moreover, GnRHa can protect ovarian function from chemotherapy-induced gonadotoxicity, an important consideration for patients desiring future fertility. Long-acting GnRHa formulations provide practical advantages without compromising efficacy, and treatment-related side effects are generally reversible and manageable [58]. Ongoing studies are aimed at refining risk stratification and optimizing the extent and duration of ovarian suppression to further enhance outcomes.

Collectively, these data emphasize that patient age represents the key determinant guiding FP strategies, influencing ovarian reserve, treatment timing, and the likelihood of success, as outlined in Table 2.

**Table 2:** Age-stratified fertility preservation strategies in women with breast cancer: biological and oncologic rationale

Age group	Oncologic context	Recommended FP approach	Biological rationale	Oncologic considerations
< 30 years	Any BC subtype	Oocyte or embryo cryopreservation (first-line)	Large follicular reserve; high AMH; low aneuploidy; high oocyte competence	Letrozole-based COS safe in HR+ disease; minimal delay to chemotherapy
	Neoadjuvant chemotherapy (urgent)	Random-start COS ± DuoStim	Maximizes oocyte yield in limited time window	Does not significantly delay systemic therapy
	HR+ disease with long endocrine therapy	Oocyte/embryo cryopreservation	Long reproductive window despite treatment delay	Pregnancy often postponed; early FP recommended
	BRCA1/2 pathogenic variant carriers	Oocyte/embryo cryopreservation preferred	Possible DNA repair impairment; still high reserve	PGT-M possible; caution with OTC
	High gonadotoxic regimens	GnRHa co-treatment	Greater ovarian resilience; temporary ovarian quiescence	Reduces POI risk but does not replace FP
30–35 years	Any BC subtype	Oocyte or embryo cryopreservation	Moderate–high reserve; early decline in oocyte competence after 35	Timing of FP remains critical
	Neoadjuvant chemotherapy	Random-start COS ± DuoStim	Efficient oocyte retrieval within limited time	Requires coordination to avoid delays
	HR+ disease with long endocrine therapy	Oocyte/embryo cryopreservation	Declining oocyte competence	Pregnancy often delayed 5–10 years
	BRCA1/2 pathogenic variant carriers	Oocyte/embryo cryopreservation preferred	Possible reduced ovarian reserve	PGT-M possible; caution with OTC
	High gonadotoxic regimens	GnRHa co-treatment	Partial ovarian protection	Adjunct, not alternative to FP
35–40 years	Any BC subtype	Embryo cryopreservation (preferred if partner available)	Reduced reserve; increased aneuploidy; more oocytes needed per live birth	Urgent referral essential; multiple cycles often required
	Neoadjuvant chemotherapy	Random-start COS; dual trigger	Optimizes yield despite time constraints	Coordination to avoid treatment delay
	HR+ disease requiring prolonged therapy	Immediate FP before endocrine treatment	Aging during therapy narrows reproductive window	Ovarian recovery ≠ preserved fertility
	BRCA1/2 pathogenic variant carriers	Individualized FP strategy	Further compromised reserve possible	Careful risk–benefit evaluation
	High gonadotoxic regimens	GnRHa co-treatment	Limited protective effect	Does not compensate for reduced reserve
> 40 years	Any BC subtype	Individualized counseling; embryo cryopreservation if feasible	Critically diminished ovarian reserve; high aneuploidy	Limited biological benefit; realistic expectations required
	Neoadjuvant chemotherapy	FP rarely feasible	Very low oocyte yield	Prioritize oncologic treatment
	HR+ disease with long endocrine therapy	Careful risk–benefit discussion	Conception attempts may occur > 45 years	Very low cumulative live birth probability
	BRCA1/2 pathogenic variant carriers	Individualized approach	Already reduced reproductive potential	Consider alternative family-building strategies
	Metastatic/high relapse risk	FP generally discouraged	Limited reproductive horizon	Oncologic prognosis prioritized
All ages	HR+ disease	Letrozole-based COS	Suppresses estradiol without reducing oocyte yield	No increased recurrence risk observed
	Prepubertal girls	Ovarian tissue cryopreservation	Only available FP option; high follicular density in young tissue	No delay to chemotherapy
	OTC in adults	Selected cases (30–40 years ideal range)	Preserves endocrine function; variable fertility outcome	Not recommended > 45 years
	IVM (selected urgent cases)	Experimental option	Avoids full stimulation; limited data	Lower pregnancy rates

*AMH* anti-Müllerian hormone, *AFC* antral follicle count, *BC* breast cancer, *BRCA* breast cancer gene, *COS* controlled ovarian stimulation, *FP* fertility preservation, *GnRHa* gonadotropin-releasing hormone agonist, *HR* + hormone receptor–positive, *IVM* in vitro maturation, *OTC* ovarian tissue cryopreservation, *POI* premature ovarian insufficiency, *PGT-M* preimplantation genetic testing for monogenic diseases

## The central role of age in fertility outcomes after cancer treatment

Patient age is one of the most decisive determinants shaping the impact of cancer treatments on ovarian reserve and long-term fertility outcomes. Younger women typically begin therapy with a larger primordial follicle pool, providing partial protection against gonadotoxicity. The study by Mordenfeld Kozlovsky et al. confirms that younger patients face a markedly lower risk of developing POI from equivalent treatment regimens [59]. However, age-related delays in conception, often caused by extended adjuvant endocrine therapy lasting 5–10 years, can offset this biological advantage. Consequently, many patients diagnosed in their early 30s attempt conception only in their late 30s or early 40s, when natural fertility has already declined substantially [60]. This temporal shift critically limits reproductive outcomes, even in women whose ovarian function recovers after treatment. Findings from the POSITIVE trial further underscore this complexity. Although 94% of women aged ≤ 42 years who experienced amenorrhea resumed menstruation within 12 months, 43% still required ART to achieve pregnancy. Thus, menstrual recovery does not necessarily equate to restored fertility. While age was not independently predictive of menses recovery in multivariable analysis, likely due to the younger average trial population, it remains the dominant factor in FP success when examined across broader cohorts [60]. This distinction becomes particularly relevant in older premenopausal patients, in whom ovarian endocrine activity may resume while reproductive capacity remains biologically constrained by age-related oocyte depletion and reduced oocyte competence. Therefore, ovarian function recovery should be interpreted as a marker of hormonal activity rather than definitive evidence of preserved fertility. Age also constrains the FP window. COS, oocyte cryopreservation, embryo cryopreservation, and OTC all yield significantly better outcomes in women under 35. In contrast, older premenopausal women face reduced ovarian response, fewer retrievable oocytes, and lower fertilization success, making age both a biological and practical barrier to preservation. These findings reinforce the necessity of early, age-informed fertility counseling and intervention [59]. Even among younger women, age moderates the degree of gonadotoxicity. Patients under 40 generally have higher baseline ovarian reserve, offering some resilience to chemotherapy. Significant declines in AMH levels after treatment demonstrate that chemotherapy can still drastically impair fertility potential [61]. Meta-analyses indicate that over 50% of young BC survivors exhibit AMH levels consistent with diminished ovarian reserve 12–24 months post-treatment. Data from the Memorial Sloan Kettering Cancer Center showed that the average age at BC diagnosis among women pursuing FP was 34 years, and 32.8 years among those completing cryopreservation [62]. Those who preserved fertility early achieved the highest post-treatment family-building success underscoring that biological opportunity, dictated by age, is often the determining factor. Importantly, younger women with germline BRCA1/2 pathogenic variants may experience an even faster decline in ovarian reserve due to impaired DNA repair, warranting early AMH testing and prompt referral for preservation. Finally, age modifies ovarian recovery trajectories: older premenopausal women are far more likely to experience irreversible ovarian failure, while younger patients may regain partial or full function albeit within a shortened reproductive window. Hormonal suppression, cancer subtype, and treatment duration further interact with age, reinforcing the need for personalized, age-stratified fertility risk assessment [62]. Beyond evidence derived from randomized clinical trials, real-world observational studies and registry-based analyses further strengthen the clinical relevance of an age-centered oncofertility framework. Population-level data demonstrate that FP uptake is strongly age-dependent, with younger women significantly more likely to undergo oocyte or embryo cryopreservation compared to women approaching 40 years of age [63]. In contrast, older reproductive-age patients show lower referral rates and reduced utilization of FP strategies, reflecting both diminished ovarian reserve and perceived lower probability of success.

Registry-based studies also confirm that ovarian function recovery following chemotherapy is markedly influenced by age. Younger patients are more likely to resume menstruation and maintain endocrine function, whereas women over 35–40 years exhibit higher rates of persistent amenorrhea and POI [64]. Importantly, real-world reproductive outcomes parallel these biological differences: cumulative pregnancy and live birth rates decline progressively with increasing age at diagnosis, even among patients who experience menstrual recovery [65]. This discrepancy highlights that restoration of menses does not necessarily translate into preserved fertility potential.

Collectively, the convergence of randomized trials and real-world evidence consistently supports patient age as the dominant modifier of FP uptake, ovarian resilience to systemic therapy, and long-term reproductive success in BC as reported in Table 3.

**Table 3:** Age-stratified impact on fertility-related outcomes in breast cancer patients

Age group	Baseline ovarian reserve (AMH/AFC)	Risk of TRA/POI after chemotherapy	Ovarian function recovery	Post-treatment pregnancy probability	FP success (oocyte/embryo cryopreservation)	Type of evidence
< 30 years	High AMH, high AFC	Low–moderate TRA risk; POI ~ 15–20%	High recovery (> 70% menses resumption within 1–2 years)	Highest spontaneous conception rates	Highest oocyte yield; best live birth probability	Observational cohorts; systematic reviews [22, 31, 61]
30–35 years	Moderate AMH, high AFC	Intermediate TRA risk	Good recovery but reduced AMH post-treatment	Often delayed by endocrine therapy	Good FP outcomes if performed early	Cohorts; registry data; meta-analyses [20, 59, 62]
36–40 years	Reduced AMF: reduced AFC	High TRA risk; POI 30–50%	Partial recovery; diminished reserve persists	Frequently require ART	Reduced oocyte yield; more cycles needed	Clinical trials, real-world [36, 60]
> 40 years	Low AMH, low AFC	Very high TRA risk; POI > 50%	Limited recovery; often irreversible	Low spontaneous pregnancy rates	Poor FP outcomes; limited efficacy	Real-world studies; registry data [22, 46, 62]

*AMH* anti-Müllerian hormone, *AFC* antral follicle count, *ART* assisted reproductive technologies, *FP* fertility preservation, *POI* premature ovarian insufficiency, *TRA* treatment-related amenorrhea

## Clinical counseling and practice guidelines

International guidelines recommend fertility counseling for all premenopausal women with cancer [59, 62]. The American Society of Clinical Oncology (ASCO) and the European Society for Medical Oncology (ESMO) both underscore the obligation of healthcare providers to discuss the potential gonadotoxic effects of cancer therapy and offer timely referral to reproductive specialists [5]. Nevertheless, younger patients are more likely to be informed and referred to reproductive specialists, often due to clinician assumptions about childbearing priorities based on age. Physicians frequently underestimate fertility concerns in older reproductive-age women, leading to under-referral and missed opportunities for preservation [66]. Data from Memorial Sloan Kettering Cancer Center shows that early referral facilitated by fertility nurse specialists significantly increases the uptake of FP services. In this model, patients received individualized education about available options, helping bridge knowledge gaps and enhance decision-making confidence. Emerging programs integrating fertility navigators and multidisciplinary care teams have demonstrated improved uptake of fertility services and reduced decision regret among patients [62]. In addition, the implementation of electronic medical record prompts and institutional protocols has been shown to improve consistency in counseling and referral practices. Tailored counseling should consider age, reproductive goals, relationship status, parity, and socioeconomic background to ensure equitable access to FP options. Financial barriers remain a major deterrent, with many women citing cost and lack of insurance coverage as reasons for forgoing FP [67]. Moreover, early integration of reproductive discussions into oncology care, ideally at the time of diagnosis or surgical consultation, can normalize fertility as a component of survivorship planning. This approach reduces barriers to timely intervention and affirms fertility-related decision-making as a legitimate and supported part of the cancer care continuum. Currently, a notable and positive change in the rate of fertility counseling at diagnosis can be observed compared to the past. One study reported that only 26% of young BC patients had documented fertility counseling in their medical records between 2006 and 2014 [68], while another study based on patient recall indicated a counseling rate of 68% during 2006–2012 [69]. More recent data from a 2015–2020 Canadian survey showed improved counseling rates, with 84% of BC patients receiving fertility discussions and strong concordance between patients and surgeons [70]. Reducing disparities across treatment centers, addressing potential age-related bias, and implementing uniform FP coverage at the state level are essential measures to improve access and equity in oncofertility counseling and services [71].

## Conclusions

**Fig. 3:**
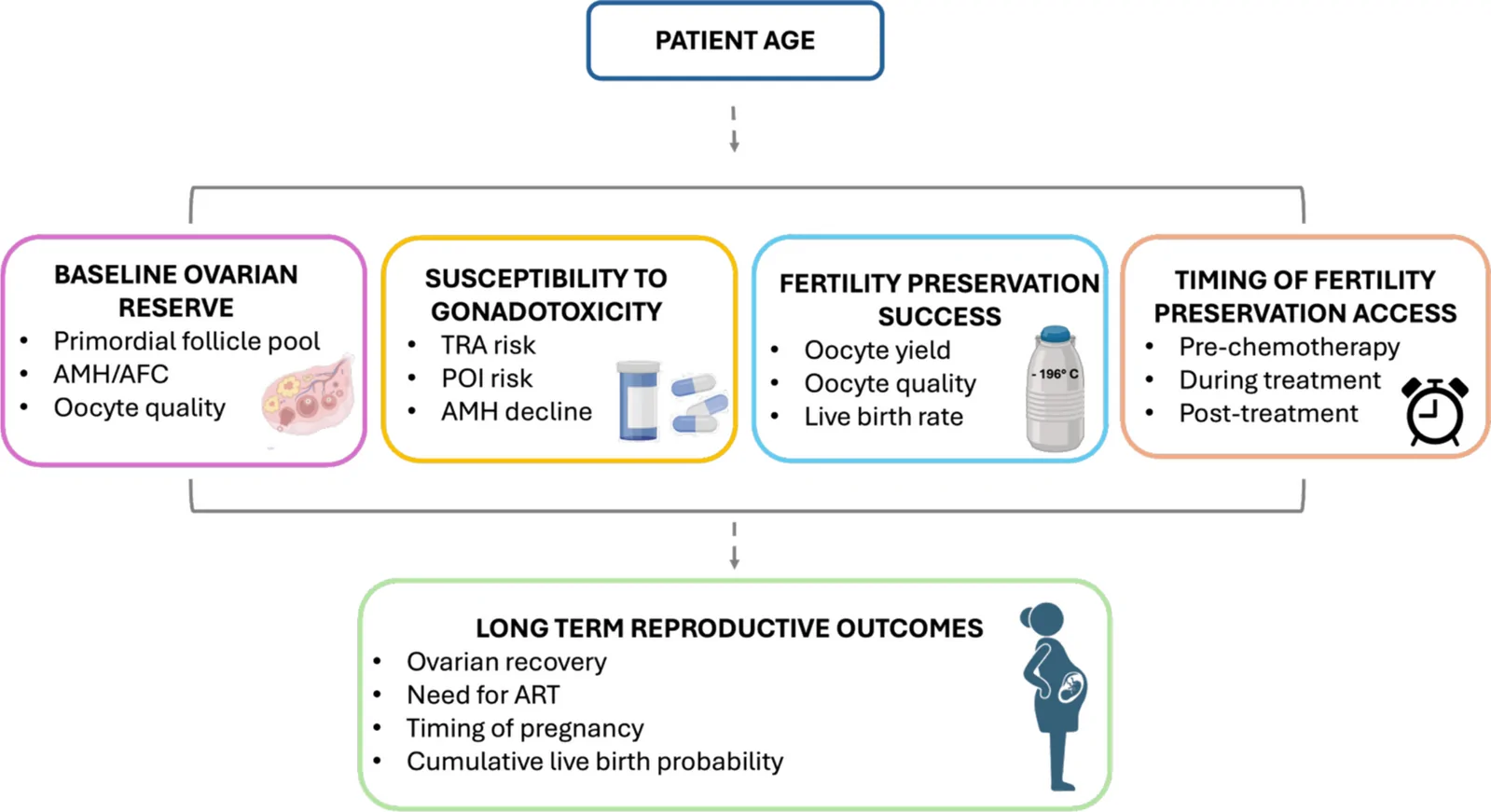
Age-centered framework of reproductive outcomes in breast cancer patients. Patient age is the primary determinant influencing baseline ovarian reserve, susceptibility to treatment-related gonadotoxicity, and fertility preservation success. These interconnected factors ultimately shape long-term reproductive outcomes, including ovarian recovery, need for ART, and cumulative live birth probability. The timing of access to fertility preservation acts as an upstream modifier, influencing the feasibility and effectiveness of available strategies, with earlier access associated with improved reproductive outcomes. AMH, anti-Müllerian hormone; AFC, antral follicle count; ART, assisted reproductive technologies; FP, fertility preservation; POI, premature ovarian insufficiency; TRA, treatment-related amenorrhea

Patient age emerges as the pivotal determinant of reproductive outcomes in women with BC, shaping both the biological resilience of ovarian reserve and the effectiveness of FP strategies. Younger patients not only possess greater ovarian capacity to withstand gonadotoxic therapies but also benefit most from timely FP interventions. However, advancing age, delayed conception due to prolonged endocrine therapy, and accelerated ovarian aging in BRCA1/2 pathogenic variants carriers collectively narrow the reproductive window, emphasizing the urgency of early and individualized counseling. Integrating patient age into every stage of FP, from risk assessment and treatment selection to timing and follow-up, should be regarded as a cornerstone of modern oncofertility care. A multidisciplinary, age-informed approach can bridge the gap between cancer survivorship and reproductive autonomy, ensuring that FP becomes a standard, equitable, and personalized component of BC management. Importantly, the distinction between ovarian endocrine recovery and actual reproductive capacity should be emphasized in clinical practice, as menstrual resumption does not reliably predict the likelihood of successful conception, particularly in older premenopausal women (Fig. 3).

## Data Availability

No datasets were generated or analysed during the current study.
